# The identification of neutrophils-mediated mechanisms and potential therapeutic targets for the management of sepsis-induced acute immunosuppression using bioinformatics

**DOI:** 10.1097/MD.0000000000024669

**Published:** 2021-03-26

**Authors:** Fang Chen, Chunyan Yao, Yue Feng, Ying Yu, Honggang Guo, Jing Yan, Jin Chen

**Affiliations:** a Nursing Department, Zhejiang Hospital; b Institute of Health Food, Zhejiang Academy of Medical Sciences; c Radiology Department, Zhejiang Hospital; d Zhejiang Experimental Animal Center, Zhejiang Academy of Medical Sciences; e Intensive Care Unit, Zhejiang Hospital; f General Practice Department, Zhejiang Hospital, Hangzhou, Zhejiang, China.

**Keywords:** Annexin A1, immunotherapy, interleukin-15, neutrophils infiltration, sepsis-induced immunosuppression, short time-series expression miner

## Abstract

Neutrophils have crucial roles in defensing against infection and adaptive immune responses. This study aimed to investigate the genetic mechanism in neutrophils in response to sepsis-induced immunosuppression.

The GSE64457 dataset was downloaded from the Gene Expression Omnibus database and the neutrophil samples (D3-4 and D6-8 post sepsis shock) were assigned into two groups. The differentially expressed genes (DEGs) were identified. The Short Time-series Expression Miner (STEM) clustering analysis was conducted to select the consistently changed DEGs post sepsis shock. The overlapping genes between the DEGs and the deposited genes associated with immune, sepsis, and immunosuppression in the AmiGO2 and Comparative Toxicogenomics Database were screened out and used for the construction of the protein–protein interaction (PPI) network. The expression of several hub genes in sepsis patients was validated using the PCR analysis. The drugs targeting the hub genes and the therapy strategies for sepsis or immunosuppression were reviewed and used to construct the drug–gene–therapy–cell network to illustrate the potential therapeutic roles of the hub genes.

A total of 357 overlapping DEGs between the two groups were identified and were used for the STEM clustering analysis, which generated four significant profiles with 195 upregulated (including annexin A1, *ANXA1*; matrix metallopeptidase 9, *MMP9*; and interleukin 15, *IL-15*) and 151 downregulated DEGs (including, *AKT1*, IFN-related genes, and HLA antigen genes). Then, a total of 34 of the 151 downregulated DEGs and 39 of the 195 upregulated DEGs were shared between the databases and above DEGs, respectively. The PPI network analysis identified a downregulated module including IFN-related genes. The deregulation of DEGs including *AKT1* (down), IFN-inducible protein 6 (*IFI6*, down), *IL*-15 (up), and *ANXA1* (up) was verified in the neutrophils from patients with sepsis-induced immunosuppression as compared with controls. Literature review focusing on the therapy showed that the upregulation of *IL*-15, *IFN*, and *HLA* antigens are the management targets. Besides, the *AKT1* gene was targeted by gemcitabine.

These findings provided additional clues for understanding the mechanisms of sepsis-induced immunosuppression. The drugs targeting *AKT1* might provide now clues for the management strategy of immunosuppression with the intention to prevent neutrophil infiltration.

## Abstract 2

Supplemental Digital Content is available in the text

**Highlights**

- Three hundred fifty-seven overlapping DEGs were identified in the neutrophil samples at D3-4 and D6-8 post sepsis.
- STEM clustered DEGs with sustained up- and down-regulated expression profiles.
- Neutrophil HLA antigen genes, IFN-related genes, and *AKT1* were downregulated.
- Neutrophil *MMP8*/9, *NFKBIA*, and *ANXA1* were upregulated in patient with immunosuppression.

## 1 Introduction

Septic syndrome (SS) represents a primary cause of mortality in critically ill patients in intensive care units (ICU). SS is mainly caused by bacteria infection, and *candidemia* is the primary cause of sepsis.^[1,2]^ It has been reported that almost 98% of patients with *candidemia* showed SS.^[1]^ In addition, the all-cause mortality at 30 days or in-hospital in patients with SS or *candidemia* was high, from 30% in sepsis to 65% to septic shock,^[1]^ with an averaged mortality of 36% to 87% at 30 days.^[1–5]^

Individuals with SS are characterized by severe disturbed immune homeostasis or suppressive immunophenotype.^[6,7]^ Immunosuppression in sepsis patients with *Candida* infection is characterized by T cell exhaustion and a concomitant decrease in positive co-stimulatory molecules, including CD28 and major histocompatibility complex, class II (HLA)-antigens.^[7,8]^ Immunosuppression intensity and duration of sepsis are positively associated with mortality and infections.^[9]^

Neutrophils are the most abundant leukocytes and have crucial roles in defensing against infection and adaptive immune responses via:

1. secretion of cytokines, including interleukin (IL)-1β and tumor necrosis factor (TNF)-α^[10]^;
2. secretion of CC and CXC chemokines and signaling mediators, such as neutrophil-derived granule contents, lipids, and reactive oxygen species (ROS)^[10–12]^;
3. cell–cell contact and communication^[13,14]^; and
4. kinases.^[15]^

A subset of human acute inflammation-responsive neutrophils could accomplish T-cell function.^[12,16]^ Wang et al showed that the increased level of neutrophil gelatinase-associated lipocalin (NGAL) was an independent risk factor for the mortality in patients with severe sepsis and septic shock.^[17]^ NGAL is secreted by activated neutrophils and various tissues in response to bacterial infections.^[18]^ The correlation of NGAL with sepsis has been evidenced by a large number of studies and clinical trials.^[19–21]^ However, there is a lack of comprehensive information on the association of neutrophils with sepsis mediated-immunosuppression and the underlying mechanism.

We performed this study to investigate the neutrophils-mediated immune responses to immunosuppression in sepsis. Sepsis-induced genetic alterations in neutrophils were investigated to uncover the underlying mechanisms of immunosuppression. Besides, the potential targets for the management of sepsis-induced immunosuppression would be identified and illustrated. This study might give us some clues about the immunosuppressive mechanism in neutrophils.

## 2 Materials and methods

### 2.1 Ethics statement

Human experiments were performed with an approval obtained from the Ethics Committee of Zhejiang Hospital, Zhejiang, China. Written informed consents were obtained from all participants before blood sampling.

### 2.2 Microarray data selection and extraction

The microarray dataset GSE64457 was selected from the public Gene Expression Omnibus (GEO) database (http://www.ncbi.nlm.nih.gov/geo/) using the searching terms of “sepsis” AND “immunosuppression” in Jan 2019. GSE64457, on the platform of GPL570 (HG-U133_Plus_2) Affymetrix Human Genome U133 Plus 2.0 Array, consisted of 23 samples, including nine neutrophil samples from patients with sepsis at D3-4 post sepsis shock, six samples at D6-8 post sepsis shock, and eight neutrophil samples from healthy controls. All the CEL data files were extracted from the GEO database for further analysis.

### 2.3 Data processing and gene expression profiling

The CEL files were processed using the Affy package (version 1.52.0, http://bioconductor.org/help/search/index.html?q=affy) for standard data normalization (MAS and quantile) and probe-symbol conversion. Probe-gene symbol conversion was conducted according to the following criteria: if multiple probes corresponded to one gene symbol, the expression value of these probes were averaged and regarded as that of the corresponding gene. Probes corresponded to none were removed. The differentially expressed genes (DEGs) in D3-4 and D6-8 samples relative to control were identified using the classical Bayesian method in the Limma package (Version 3.10.3; http://www.bioconductor.org/packages/2.9/bioc/html/limma.html),^[22]^ with the criteria of *P* value < .05 and |log_2_FC(fold change)| ≥ 1. The overlapping genes between different comparisons were identified using the Venn diagram analysis.

### 2.4 STEM clustering of DEGs expression profiling

Short time-series expression miner (STEM) clustering algorithm (version1.3.11; http://www.cs.cmu.edu/∼jernst/stem/) was used to perform the clustering of the time series DEGs based on the changed expression patterns. The STEM clusters or profiles of the DEGs with similar expression profiling at control, D3-4, and D6-8 after sepsis were identified following the criteria of minimum correlation coefficient > .7, *P* < .05, and ≥ 20 DEGs.

### 2.5 Gene set enrichment analysis

Gene set enrichment analysis was performed for the DEGs in significant STEM profiles. The Gene Ontology (GO)^[23]^ biological processes and Kyoto Encyclopedia of Genes and Genomes (KEGG) pathways^[24]^ significantly associated with the DEGs were identified with the criteria of *P* < .05 and count ≥2. All analyses were performed using the common enrichment analysis tool DAVID (version 6.8, https://david.ncifcrf.gov/).^[25]^

### 2.6 Selection of the hub genes associated with the immunosuppression and sepsis

To identify the genes that may have important roles in sepsis-induced acute immunosuppression, the genes have been recognized to be associated with the immune, sepsis, and immunosuppression were screened out from the online databases including AmiGO2 (http://amigo.geneontology.org/amigo) and Comparative Toxicogenomics Database (CTD, 2020 update; http://ctd.mdibl.org/). The two databases provide valuable references to the association of genes and pathways with diseases. The immune-related genes were selected in AmiGO2 using the search term of “immune,” and the genes related to “immune suppression” and “sepsis” were identified in the CTD. The shared genes between the DEGs and the obtained genes in the above databases were identified and used as candidates for further analysis. Besides, the KEGG pathways related to “sepsis” were also identified from the CTD.

### 2.7 PPI network analysis

The above shared genes were used to construct the protein–protein interaction (PPI) network to analyze the interaction characteristics among genes. The interactions between the gene products were identified from the String database (version 10.0, https://string-db.org; interaction score=0.4).^[26]^ The visualization of the PPI network was implemented using the Cytoscape (version 3.2.0, http://www.cytoscape.org/).^[27]^ Next, the modules in the PPI network were identified using MCODE plugin MCODE (Version1.4.2, http://apps.cytoscape.org/apps/MCODE)^[28]^ in Cytoscape with a score >5. Then, DEGs in the modules were employed to identify the GO biological processes and KEGG pathways (*P* < .05 and count ≥ 2) using the DAVID tool.

### 2.8 Selection of the potential targets for the therapy of immunosuppression

There is emerging evidence showing the efficacy of immunological modification therapies on sepsis or sepsis-induced immunosuppression.^[8,29–31]^ Therapy strategies were selected from literature, and the potential targets of them reported in literature would be identified. The potential drugs targeting the DEGs in sepsis-induced immunosuppression were predicted in the Drug–Gene Interaction database 2.0 (DGIdb2.0; http://www.dgidb.org/) with the reset filters of “FDA approved” and “Immunotherapies.” Only drugs with accurate definitions including immunotherapy, agonist, inducer, inhibitor, and antagonist are retained. In addition, the genes expressed by the immune cells (including neutrophils, Treg cells, macrophages, natural killer [NK] cells, dendritic cells [DC], T cell, and B cell) related to sepsis or sepsis-induced immunosuppression were identified from the CTD database (2020 update). The shared genes between immune cell-related genes and DEGs were retained and used for the construction of the drug–gene–therapy–cell network.

### 2.9 Patient subject and sample collection

Six patients with sepsis-induced immunosuppression (male = 4 and female = 2, aged 62.6 ± 8.3 years old) were collected from the Department of ICU, Zhejiang Hospital, Hangzhou, China, between February and August 2019. Six sex- and age-matched healthy controls (male = 4, female = 2; 65.1 ± 7.2 years old), without known diseases, were enrolled and used as controls. Septic shock was defined according to the diagnostic criteria of the Third International Consensus Definitions for Sepsis and Septic Shock,^[32]^ and patients were included in they met at least two of the following criteria:

1. temperature >38°C or <36°C;
2. heart rate >90 beats/min;
3. respiratory rate >20 breaths/min; and
4. white blood cell count >12 × 10^9^ cells/L or < 4 × 10^9^ cells/L, or immature neutrophils >10%.

The peripheral blood samples were collected from patients at D3-4 and D6-8 post sepsis shock and all healthy controls. The neutrophils samples were purified according to the instruction from a Human Neutrophil Isolation Kit (Solarbio, Beijing, China) and stored at −20°C before analysis.

### 2.10 Real-time PCR analysis

The total RNA was extracted from neutrophils using the TRIzol reagent (Qiagen, Hilden, Germany). RNA reverse transcription and first-strand DNA synthesis were performed with a High-Capacity cDNA Reverse Transcription kit (Life Technologies Corporation, Carlsbad, CA) and a first-stand cDNA synthesis kit (Invitrogen, Carlsbad, CA). The specific PCR primer pairs are: *ANXA1* forward primer 5’-GCAGGCCTGGTTTATTGAAA-3’ and reverse primer 5’-GCTGTGCATTGTTTCGCTTA-3’; *SIPR1* forward primer 5’-GGATTGGTTATTGGAGTGTTT-3’, reverse primer 5’-CATATTTTCTAAATTTTTATTTACCTC-3’; *EDN1* forward primer 5’-CCAAGGAGCTCCAGAAACAG-3’, reverse primer 5’-GATGTCCAGGTGGCAGAAGT-3’; *RSAD2* forward primer 5’-CAGCGGGAAACGAAAGCGAA-3’, reverse primer 5’-AGAACCTCACCAACTTGCCC-3’; *IFI6* forward primer 5’-TCTTCACTTGCAGTGGGGTG-3’, reverse primer 5’-ATACTTGTGGGTGGCGTAGC-3’; *CD74* forward primer 5’-GGCAACATGACAGAGGACCA-3’, reverse primer 5’-TCCAAGGGTGACGAAAGAGC-3; *AKT1* forward primer 5’-CTGCACAAACGAGGGGAGTA-3’, reverse primer 5’-GCGCCACAGAGAAGTTGTTG-3’; *IL-15* forward primer 5’-TGTTTCAGTGCAGGGCTTC-3’, reverse primer 5’-TTCCTCACATTCTTTGCATCC-3’; β-*actin* forward primer 5’-AGAGGGAAATCGTGCGTGAC-3’, reverse primer 5’-CAATAGTGATGACCTGGCCGT-3’. PCR amplification was conducted using a Bestar Sybr Green qPCR master mix kit (DBI Bioscience, Shanghai, China). The relative expression levels of genes were calculated using the 2^−ΔΔCt^ methods.

### 2.11 Statistical analysis

All data are expressed as the mean ± standard deviation. Differences in the expression of mRNAs among three groups were analyzed using the one-way ANOVA test. A *P* value < .05 was considered statistically significant.

## 3 Results

### 3.1 DEG identification

**Figure 1:**
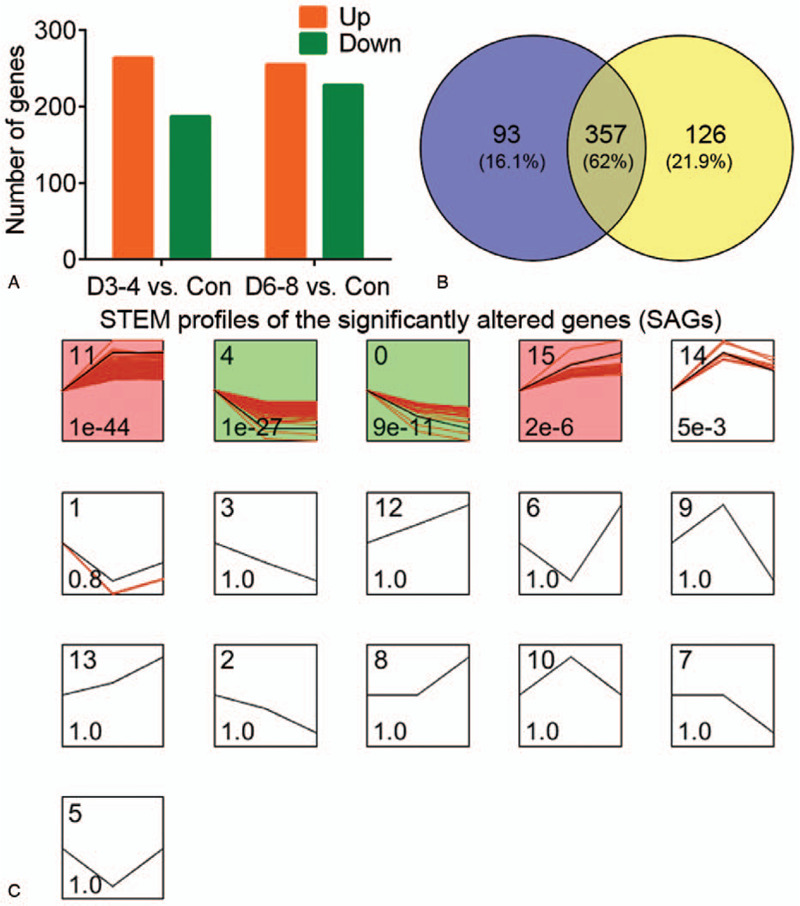
The statistics and clustering analysis of differentially expressed genes (DEGs) in neutrophils from septic patients. (A) The number of DEGs in the neutrophil samples from patients at D3-4 and D6-8 post sepsis shock. (B) The Venn diagram of DEGs. (C) The STEM profiles of DEGs. STEM time series are set as control, D3-4 and D6-8. Green and red profiles note up-and down-regulated DEGs in significant profiles (with *P* < .05, correlation coefficient > .7 and gene number ≥20), respectively.

After data normalization, a total of 450 DEGs and 483 DEGs were identified from the neutrophil samples at D3-4 and D6-8 post sepsis shock as compared with controls, respectively (Fig. 1A). Besides, 357 overlapping DEGs (62%) between the 450 and 483 DEGs at the two time intervals were identified using the Venn diagram analysis (Fig. 1B).

### 3.2 STEM clusters of the DEGs

Figure 1C shows the STEM analysis identified 16 profiles, including four significant profiles (with *P* < .05 and correlation coefficient > .7, with ≥20 genes). The DEGs in the profile 0 (0.0, −2.0, −3.0) were gradually decreased at D3-4 and D6-8 post sepsis shock, while the DEGs in the profile 15 (0.0, 2.0, 3.0) were gradually increased. The DEGs in the profile 4 (0.0, −1.0, −1.0) and 11 (0.0, 1.0, 1.0) were dysregulated at the first 3 to 4 days post sepsis shock, and kept at the same level till the 6 to 8 days post sepsis shock. According to the expression profiles, the DEGs in the profiles 0 and 4 were sorted into the green clusters (down-regulation, n = 151; supplementary table S1, http://links.lww.com/MD/F950), while the DEGs in the profiles 11 and 15 were sorted into the red clusters (up-regulation, n = 195; supplementary table S1, http://links.lww.com/MD/F950).

### 3.3 Gene set enrichment for DEGs in the green and red clusters

**Figure 2:**
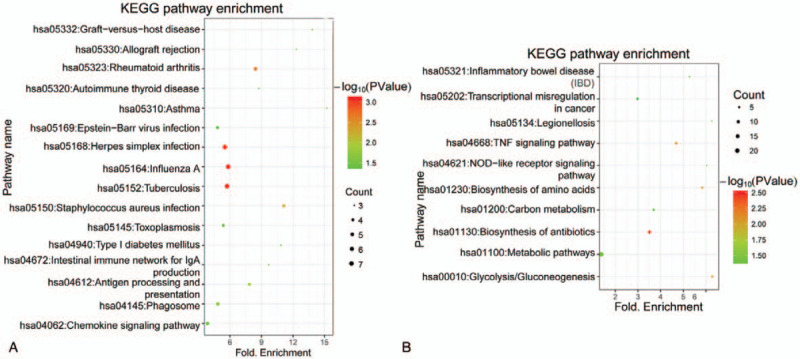
The KEGG pathways associated with differentially expressed genes (DEGs) in the green (A) and red (B) cluster. The color circle represents the number of genes involved in the corresponding KEGG pathway. The larger the circle, the higher the gene number. Green color notes p values closer to 0. The redder, the higher the *P* value.

The functional enrichment analysis showed that the DEGs in the green clusters were associated with the GO biological processes including “GO:0060337: type I interferon signaling pathway,” “GO:0006954: inflammatory response,” “GO:0006955: immune response,” and “GO:0045087: innate immune response” (supplementary table S2, http://links.lww.com/MD/F951). KEGG pathway enrichment analysis showed that the downregulated DEGs were mainly involved in “hsa05164: Influenza A,” “hsa05323: Rheumatoid arthritis,” “hsa05330: Allograft rejection” and hsa04672: Intestinal immune network for IgA production” (Fig. 2A).

The DEGs in the red cluster were associated with GO biological processes including “GO:0006096: glycolytic process,” “GO:0006954: inflammatory response,” “GO:0006809: nitric oxide biosynthetic process,” and “GO:0042542: response to hydrogen peroxide” (supplementary table S3, http://links.lww.com/MD/F952), and were involved in the KEGG pathways including “hsa01130: Biosynthesis of antibiotics,” “hsa00010: Glycolysis/Gluconeogenesis,” “hsa04668: TNF signaling pathway,” “hsa04621: NOD-like receptor signaling pathway,” and “hsa05321: Inflammatory bowel disease” (Fig. 2B).

### 3.4 Selection of the hub genes in green and red clusters

The searching in the AmiGO2 database generated 3276 immune-related genes, and the searching in the CTD produced 11,300 immune suppression-associated genes and 22,525 sepsis-related genes, respectively. After overlapping with the DEGs in the above green and red clusters, 34 downregulated DEGs were shared between the databases and green clusters (0 and 4) and 39 upregulated DEGs were shared between the databases and red clusters (11 and 15), respectively (supplementary table S4, http://links.lww.com/MD/F953).

### 3.5 PPI network analysis for the DEGs in the green and red clusters

**Figure 3:**
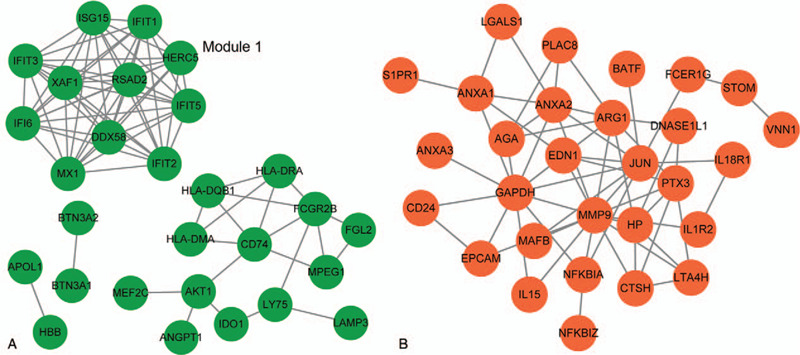
The protein–protein interaction (PPI) network of the upregulated and downregulated hub genes. (A) and (B) The PPI network was constructed using the differentially expressed genes (DEGs) in the green (n = 28) and orange (n = 29) STEM clusters, respectively. One module was found in the PPI network of the downregulated genes using the MCODE plugin in Cytoscape. Green and red notes downregulated and upregulated expression in both D3-4 and D6-8 neutrophils in patients post sepsis shock.

The PPI network of the DEGs in the green and red clusters was comprised of 28 nodes (gene products) and 77 lines (interactions; Fig. 3A), and 29 nodes and 60 lines, respectively (Fig. 3B). Forty-nine GO functional categories including “GO:0006955: immune response,” “GO:0006952: defense response,” “GO:0009615: response to virus,” and “GO:0043067: regulation of programmed cell death” enriched the downregulated DEGs in the green clusters, including interferon (IFN)-inducible protein 6/mitochondrial antiapoptotic protein G1P (*IFI6*), DEAD-box helicase 58 (*DDX58*), radical S-adenosyl methionine domain containing protein 2 (*RSAD2*) gene, and IFN-stimulated gene 15 (*ISG15*; supplementary table S5, http://links.lww.com/MD/F954). Besides, one cluster of genes (including *AKT1*, *CD74*, and *IFI6*) were associated with “GO:0042981: regulation of apoptosis” and “GO: 0043067:regulation of programmed cell death.” One module with a score of 11.00 consisting of 11 genes and 55 interactions was identified from the PPI network of the downregulated DEGs (green; Fig. 3A). Each of the nodes interacted with the other 10 nodes in the module. Of these downregulated genes in this module, six IFN-related genes, including *ISG15*, *IFI6*, IFN-induced protein with tetratricopeptide repeats (*IFIT*)1, *IFIT2*, *IFIT3*, and *IFIT5*, were included.

Also, enrichment analysis showed that the biological processes including “GO:0006955: immune response,” “GO: 0006952:defense response,” “GO:0042127: regulation of cell proliferation,” “GO: 0042981: regulation of apoptosis,” and “GO:0043067: regulation of programmed cell death” enriched the upregulated DEGs in the red clusters, including annexin A1 (*ANXA1*), *IL-15*, *CD24*, sphingosine-1-phosphate receptor 1 (*S1PR1*), *JUN*, endothelin 1 (*EDN1*), among others.

### 3.6 Characterization of the downregulated and upregulated DEGs via network

**Figure 4:**
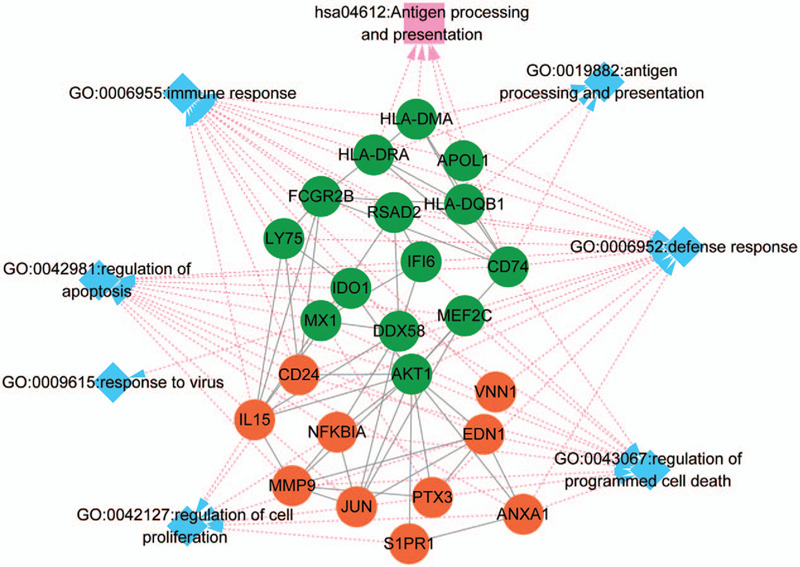
The network involving the common pathway and biological processes and differentially expressed genes (DEGs) in the network modules. The green and orange circle notes the downregulation and upregulation, respectively. The diamond indicates the biological processes, and the square represents the pathway. The involvement of genes in biological processes and pathways are indicated by dotted lines.

The gene-biological process network involving the upregulated and downregulated DEGs with similar biological processes is shown in Figure 4. Here, we show that a cluster of downregulated genes including major histocompatibility complex, class II, DM alpha (*HLA*-*DMA*), DR alpha (*HLA*-*DRA*), DQ beta 1 (*HLA-DQB1*), and CD74 (*HLADG*) are involved in the “hsa04612: Antigen processing and presentation” and the “GO: 0019882: antigen processing and presentation.” These factors were associated with “GO: 0006955: immune response” and “GO: 0006952: defense response” directly or indirectly via interacting with the Fc fragment of IgG receptor IIb (*FCGR2B*) gene (Fig. 4). The “GO:0043067: regulation of programmed cell death” and “GO:0042981: regulation of apoptosis” biological processes enriched the upregulated genes including *EDN1*, *ANXA1*, nuclear factor κB (NF-κB) inhibitor alpha (*NFKBIA*), *IL-15*, and matrix metallopeptidase 9 (*MMP9*), and the downregulated genes including *IFI6*, *AKT1*, and *CD74*. These results showed that the DEGs in sepsis-induced immunosuppression play important roles in immune and defense responses as well as in cell proliferation, programmed cell death, and apoptosis.

### 3.7 PCR validation of the expression profiling of several DEGs in human neutrophils

**Figure 5:**
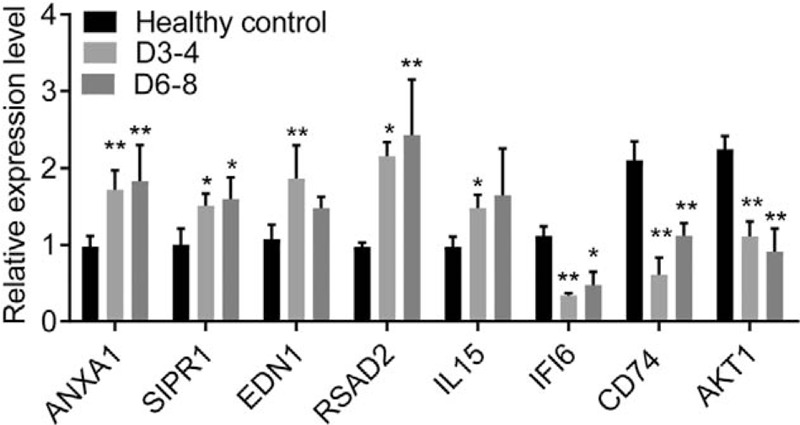
The expression profiling of eight randomly selected genes in the neutrophil samples. The neutrophil samples were isolated from the peripheral blood samples from six patients with sepsis-induced immunosuppression (at D3-4 and D6-8 post sepsis shock) and six sex/age-matched healthy controls. The difference is analyzed using the non-way ANOVA test. ^∗^ and ^∗∗^ notes the significant level at *P* < .05 and .01 compared with control, respectively.

Eight genes were randomly selected from the gene-biological process network for the validation of expression profiling using the PCR analysis. PCR confirmed the significant upregulation of the genes including *ANXA1*, *SIPR1*, *EDN1*, and *RSAD2* in the neutrophils samples from patients with sepsis-induced immunosuppression (at 3–4 days and/or 6–8 days post sepsis shock) compared with controls (*P* < .05; Fig. 5). Besides, the downregulation of the *IFI6*, *CD74*, and *AKT1* genes was confirmed (Fig. 5).

### 3.8 Analysis of the therapy targets and potential mechanisms in sepsis-induced immunotherapy

**Figure 6:**
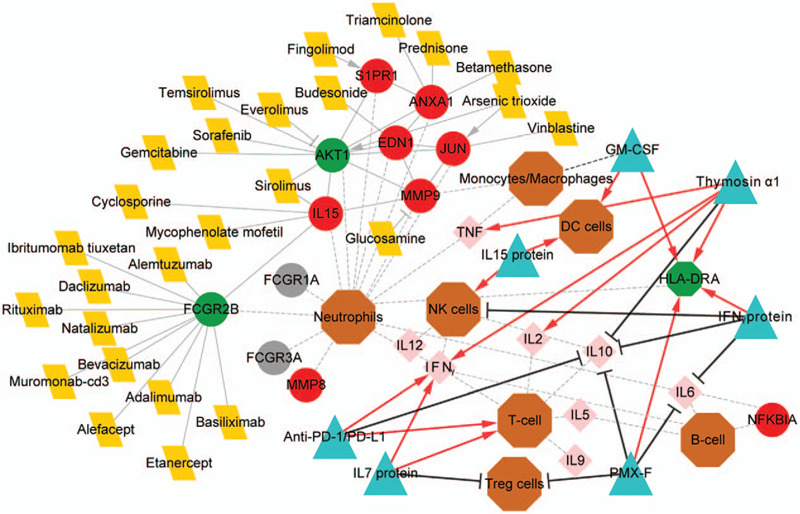
The network presenting the interactions between differentially expressed genes (DEGs), drugs, immune cells, cytokines, and therapies. The differentially expressed gene, drug, immune cell, cytokines, and therapy are indicated by circle, parallelogram, octagon, diamond, and triangle, respectively. The mechanisms underlying therapy are indicated by bold lines (red arrow lines show promotion, and black T lines show inhibition). The genes and cytokines belong to immune cells are indicated by dotted lines. The drugs with the annotation of “inducer” and “agonist” for the DEGs are noted by arrow lines, and drugs with “inhibitor” and “antagonist” are indicated by T lines. CTD = Comparative Toxicogenomics Database, DC cells = dendritic cells, GM-CSF = granulocyte-macrophage colony-stimulating factor, NK cells = natural killer cells, PD-1 = programmed cell death 1, PD-L1 = programmed cell death ligand 1, PMX-F = polymyxin B covalently immobilized on fibers.

Seven therapy strategies for sepsis or sepsis-induced immunosuppression were found by literature review (Table 1). Among these therapies, anti-PD-1 (programmed cell death 1)/PD-L1 (programmed cell death ligand 1) and IL7 promotes the proliferation of T-cell,^[8,33–36]^ the therapies including granulocyte-macrophage colony-stimulating factor (GM-CSF), polymyxin B covalently immobilized on fibers (PMX-F), IFNγ, and thymosin α1 all increases the production of HLA-DR antigens and decreases IL-10 secretion.^[8,36–41]^ Besides, we found that the IL-10, MMP7/9, GM-CSF, and other CSFs (including CSF1 and CSF2) are macrophage genes/cytokines (Table 2). IL-10 is also reported to be expressed and secreted by the T-cell, B-cell, and NK cells.^[42–44]^ The upregulated DEGs including *NFKBIA*, *MMP9*, and *MMP8* are B-cell, macrophage, and neutrophil specific gene, respectively. Moreover, IFNγ is expressed by T-cell, B-cell, neutrophil, and NK cells (Table 2). Accordingly, the network consisting of the PPI network, drug-gene interaction, and reported therapy mechanisms was constructed and shown in Figure 6.

**Table 1:** The recognized therapy strategies and targeting mechanisms for sepsis or sepsis-induced immunosuppression.

Therapy	Mechanism	Ref
Anti-PD-1/PD-L1	Promotes T-cell proliferation and IFNγ secretion, and decreases IL-10 secretion	^[8,32]^
IL-7 protein	Promotes IFNγ secretion, and T-cell proliferation, reduces Treg cells	^[8,33–35]^
IL-15 protein	Increases NK and DC cells, promotes T-cell proliferation and IFNγ secretion, inhibits neutrophil apoptosis	^[8,30,36]^
GM-CSF	Promotes HLA-DR secretion, monocytes differentiate into DC cells	^[37,38]^
IFNγ	Promotes HLA-DR secretion; decreases NK cell proliferation, IL-6 secretion and IL-10 secretion	^[35,39]^
Thymosin α1	Increases IL-12, IL-2, IFNα/γ, HLA-DR, TNF-α, and decreases IL-10	^[40,41]^
PMX-F	Increases HLA-DR, and reduces Treg cells, IL-10, and IL-6	^[8]^

CTD = comparative toxicogenomics database, DC cells = dendritic cells, GM-CSF = granulocyte-macrophage colony-stimulating factor, NK cells = natural killer cells, PD-1 = programmed cell death 1, PD-L1 = programmed cell death ligand 1, PMX-F = polymyxin B covalently immobilized on fibers.

**Table 2:** The immune cells and target genes identified in the CTD and literature.

Cell type	Related genes	CTD/Ref
Monocyte/macrophage	MMP7/9, GM-CSF, CSF1/2, TNF, IL-10	CTD^[8,42,45]^
T-cell	IL-10, IL-13, IL-5, IL-9, IL-2, TCR, IFNγ	CTD^[36]^
B-cell	NFKBIA, BCL2, IL-5, IL-6, IL-10, IFNγ	CTD^[42,46]^
Neutrophils	MMP2/7/8, FCGR3A, IFNγ, TNF, IL-6, ROS, FCGR1A	CTD^[8,31,35]^
NK cells	IFNγ, IL-12, IL-10	^[35,42–44]^

CTD =  Comparative toxicogenomics database, NK cells = natural killer cells.

In this network, the macrophage gene *MMP9* interacted with three upregulated genes including *EDN1*, *JUN*, and *IL-15*, and the downregulated *AKT1* gene, which was targeted by five drugs including gemcitabine, arsenic trioxide, and everolimus (Fig. 6 and supplementary Table S6, http://links.lww.com/MD/F955). Besides, *GM-CSF* is a macrophage gene and another CSF member, *CSF1R*, is identified to be a downregulated gene in the neutrophils in sepsis-induced immunosuppression (supplementary Table S1, http://links.lww.com/MD/F950). The downregulated neutrophil gene *FCGR2B* was targeted by 11 drugs and interacted with *IL-15* (Fig. 6). These findings showed the potential roles of these DEGs in the pathology of sepsis-induced immunosuppression or in the therapeutic management for immunosuppression.

## 4 Discussion

The crucial roles of the molecular behavior of neutrophils in immune diseases and sepsis patients are being gradually identified and unveiling.^[14,15]^ Neutrophils modulate the immunosuppression via cellular level (cell–cell contact), molecular level (cytokines, chemokines, and signaling mediators), and genetic behavior (kinases).^[10–15,45]^ Our present study suggested the global characteristics of the DEGs in neutrophils in patients with maximal sepsis-induced immunosuppression (at day 3-8 post sepsis shock). These DEGs included the upregulated *MMP8/9*, *IL-15*, *JUN*, *NFKBIA*, and *ANXA1*, and the downregulated *HLA-DR*, *AKT1*, *IFI6*, *IFITs*, *CSF1R*, and *FCGR2B*. In total, this study showed that the mechanisms underlying sepsis-induced immunosuppression were different from those reported by Demaret et al,^[46]^ who showed that the proportion of CD10 ^dim^ CD16 ^dim^ neutrophils was associated with survival of patients with sepsis. These genes are the therapy targets for sepsis or sepsis-induced immunosuppression and are involved in the biological processes including immune responses and the regulation of cell proliferation, apoptosis, and programmed cell death. Some of these genes, including CSFs, IFN related genes, HLA antigen genes, and *IL-15* have been identified as the management targets for sepsis-induced immunosuppression.^[8,33–36,39–41]^

**Figure 7:**
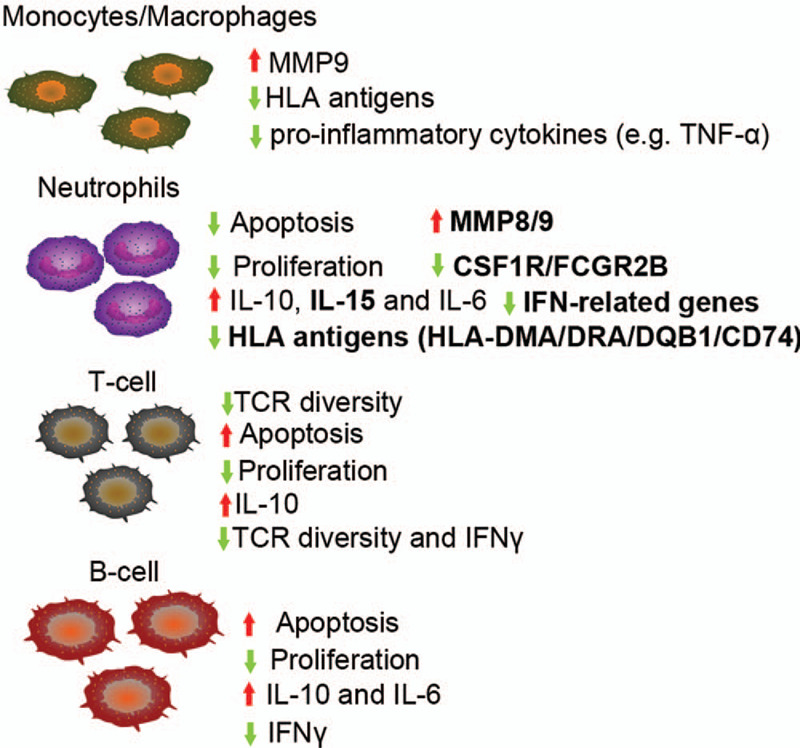
The abridged general view of sepsis-induced immunosuppression. The blue and red arrow notes the downregulation and upregulation, respectively. IFNγ = interferon γ, IL = interleukin, IL-10 = interleukin 10, MMP9 = matrix metallopeptidase 9, NK cells = natural killer cells, TCR = T cell receptor, Treg cells = T regulatory cells, TNF-α = tumor necrosis factor α. Sepsis-induced immunosuppression is characterized by increased IL-10 secretion and the apoptosis of T-cell and B-cell, enhanced proliferation of neutrophils, and decreased production of IFNγ and HLA antigens. The bold words indicate the identified features in present study using bioinformatics methods.

The mechanisms of sepsis-induced immunosuppression include the apoptosis of adaptive immune cells including the T-cell, NK cells, and B-cell, the decreased production of IFNγ and HLA-DRA, and the increased production of neutrophil immunosuppressive cytokines IL-10 and IL-6, and increased percentage of Treg cells^[8,36,39,47,48]^ (Fig. 7). The increased apoptosis of neutrophils, T cells, and B cells during immunosuppression has been reported^[8]^ (Fig. 7). IL-10 is expressed by macrophages, myeloid DCs, CD4^+^ T cells, NK cells, transforming growth factor (TGF)β-treated Treg cells, and B-cell.^[8,49,50]^ During sepsis, neutrophils are the significant producers of IL-10.^[51]^ The increased serum IL-10 by monocytes may contribute to the increased percentage of Treg cells,^[8]^ and the increased neutrophil apoptosis and NK cell cytotoxicity.^[50,52,53]^ Moreover, the production of IL-10 in NK cells and T-cell could be enhanced by IL-15, a potent T-cell stimulating factor.^[50,54]^ Park et al^[50]^ showed that IL-15 was the most potent inducer of IL-10 in human NK cells, and IL-15 showed an additive effect on IL-10-induced NK cytotoxicity. They also showed that IL-10 did not influence the production of IFN-γ or TNF-α in NK cells.^[50]^ Unlike IL-10, IL-15 protects neutrophils from apoptosis via activating the NF-κB signaling and enhances the functions of multiple innate immune cells in patients with human immunodeficiency.^[55,56]^ It also increases the secretion of macrophages cytokines, the levels of IFNγ, and the percentage of NK cells, DCs, and CD8+ T cells^[30,57,58]^ (Fig. 6). IL-15 promotes the production of MMP9 in human peripheral blood mononuclear cells (PBMCs),^[59]^ or induces macrophage infiltration in polymyositis through regulating the NF-kB signaling pathway.^[60]^*MMP9* is a macrophage gene, and its expression is upregulated in patients with sepsis.^[61]^ Our present study showed the expression of *IL-15*, *NFKBIA*, *MMP8*, and *MMP9* were significantly upregulated in the neutrophil samples from patients with sepsis-induced immunosuppression. These findings showed that the neutrophils have significant protective functions on defensing sepsis-induced immunosuppression.

During the development of sepsis, NK cells are the principal producers of IFNγ, which acts as the main activator of macrophages.^[8]^ The downregulation of *IFIT1*, whose upregulation had been reported in neutrophils from patients with antiphospholipid syndrome,^[62]^ was also confirmed in sepsis patients. Both *IFIT1* and *ISG15* are pro-inflammatory genes and are associated with viral resistance and defense in human.^[63–65]^*ISG15* is IFN-inducible, and the expression of *ISG15* prevents *IFN* amplification and auto-inflammation in turn.^[66]^*ISG15*-deficient patients with viral diseases had a high level of IFN-α/β,^[66]^ while decreased *IFIT1* expression was correlated with the increased HBV replication.^[65]^ The downregulation of IFN-induced proteins, including *IFIT1*, *IFIT2*, *IFIT3*, *IFIT5*, *ISG15*, and *IFI6*, might suggest the downregulation of IFN production. It has been reported that inhibition of *CCR3* restricted the IFN-γ-medicated changes in *MCP*-3, *MIP*-5, and *RANTES* in neutrophils.^[67]^ The administration of *IFN*, or *IFN*-γ, in neutrophils subsequently activated the expression of CCR3-mediated factors and CCR3 signaling, as well as the migration of neutrophils.^[67]^ Moreover, the immunotherapies for immunosuppression including anti-PD-1/PD-L1, Recombinant IL-15, and thymosin α1 increase the production of IFNγ and decrease the secretion of IL-10 in patients, animal and cellular model of sepsis-induced immunosuppression^[8,33,36–39]^ 7. Recombinant IFNγ also recognized as immunotherapy for immunosuppression, as it promotes HLA antigen secretion and decreases NK cell proliferation, IL-6 secretion, and IL-10 secretion^[36,39]^ (Fig. 6). The downregulation of the IFN-induced genes in the neutrophils showed that neutrophils might be the target cells for the above immunotherapies. The management targeting the upregulation of these genes might of great value for preventing immunosuppression.

The immunotherapies including anti-PD-1/PD-L1, GM-CSF, thymosin α1, recombinant IFNγ, and PMX-F all increase the production of HLA-DRA and decrease the secretion of IL-10 in patients, animal and cellular model of sepsis-induced immunosuppression.^[8,33,36–39]^ Anti-PD-1/PD-L1, IL-7 protein, and Thymosin α1 immunotherapies also increase the secretion of IFNγ.^[8,33,7,11,12]^ In addition, the IL-15 protein shows potential therapeutic efficacy in immunosuppression as it promotes T-cell proliferation and IFNγ secretion and inhibits neutrophil apoptosis.^[30]^ Our present study showed that four HLA antigens, including *HLA*-*DMA*, *HLA*-*DRA*, *HLA-DQB1*, and CD74 (*HLADG*), and six IFN-related genes, including *IFI6*, *IFIT1*/2/3/5, and *ISG15*, were downregulated in the neutrophil samples from patients with sepsis-induced immunosuppression. These findings demonstrated that neutrophils are the most extensive target cells for these therapies.

Among the sustained upregulated genes in neutrophils post sepsis, we identified *ANXA1*, *MMP9*, *SIPR1*, and *JUN* as the candidate genes. ANXA1 is a membrane adhesive and an anti-inflammatory protein that plays an important and homeostatic role in the innate immune system via Th1/Th2 shift, T-cell activation, favoring Th1,^[68,69]^ and modulation of the apoptosis, migration, and recruitment of neutrophils.^[70]^*ANXA1* prevents the recruitment of neutrophils to the inflammatory site, adhesion to endothelium, transmigration, as well as induces neutrophil apoptosis.^[70,71]^ In addition, *ANXA1* inhibited T cell proliferation and IFNγ production in human PBMCs.^[72]^*S1PR1*, which is secreted by neutrophils, is an effective mediator of S1P signaling for the activation of ERK1/2.^[73]^ It was reported that increased SIP reduced neutrophil recruitment, adhesion to endothelial cells via IL-8, and migration via enhancing endothelial barrier integrity.^[73]^ Our analysis showed that *ANXA1* and *S1PR1* interacted with the downregulated neutrophil gene *AKT1*. The overexpression of *ANXA1*, a kinase for the phosphorylation of GATA3, also promotes the production of IFN*γ* in human and murine Th2 cells,^[74]^ which is associated with the binding of GATA3 on the promoter of *ANXA1*.^[68]^ Besides, the activation of *AKT1* promotes IFN*γ* expression in the Th2 cells by preventing the posttranscriptional modification of GATA3 on *ANXA1*.^[68]^ Wang et al^[75]^ recently presented a positive activation of IL-15 induced AKT phosphorylation on the activity and survival of NK cells. Our present study showed the reverse expression profiling between the *ANXA1* (up) and *AKT1* (down) in the neutrophil samples from patients with sepsis-induced immunosuppression. Hence, the *AKT1* and *ANXA1* play important roles in the inflammatory response and proinflammatory function, which may show the clues for understanding the immunosuppression pathology. Both *ANXA1* and *S1PR1* increment or upregulation indicate the inhibition of neutrophil recruitment, adhesion to endothelial cells, and migration, suggesting the intention to prevent neutrophil infiltration.

Our drug analysis identified that gemcitabine, sorafenib, sirolimus, and arsenic trioxide are interacted drugs of AKT1, while everolimus is an inhibitor of AKT1. Everolimus is a rapamycin inhibitor and the clinical immunosuppressant used after organ transplantation.^[76,77]^ Everolimus reduces the donor-specific HLA antibodies and endothelial cell injury in heart transplants.^[78]^ Gemcitabine promotes tumor cell-derived inflammatory responses, including decreased IFN-γ-producing CD4 and CD8 T cells, and therefore resulting in immunosuppression in mouse model.^[79]^ Also, the combination of gemcitabine with rosiglitazone decreased the immunosuppression in immunocompetent animals,^[80]^ as it enhances circulating CD8+ T cells and limiting Treg cells. However, an in vitro study by Kan et al^[81]^ showed that gemcitabine suppressed the induction of Treg cells. Gemcitabine could suppress phospho-Akt expression, and the migration and invasion of pancreatic ductal adenocarcinoma cells.^[82]^ The combination of GM-CSF and gemcitabine induced a high level of immune activation and T-cell proliferation in patients with stage I or II pancreatic adenocarcinomas.^[83]^ The downregulation of sorafenib on the Akt signaling pathway has been reported in a wide range of research studies on human diseases.^[84,85]^ However, the application of these drugs for the management of sepsis-induced immunosuppression has not been reported till now. The identification of these drug-gene interactions may provide clues for the management of sepsis-induced immunosuppression.

## 5 Conclusions

In conclusion, our present study predicated the crucial roles of two cluster DEGs in neutrophils from patients with sepsis-induced immunosuppression. The upregulated DEGs including *MMP8/9* and NFKBIA and the downregulated DEGs including *AKT1*, HLA antigen genes (*HLA*-*DMA*, *HLA*-*DRA*, *HLA-DQB1*, and *CD74*/*HLADG*), and IFN-related genes (such as *ISG15*, *IFIT1*, and *IFI6*) were the potential targets for the management of immunotherapies for sepsis-induced immunosuppression. The identification of the deregulated genes including downregulated *AKT1* and upregulated *ANXA1* and *S1PR1* showed additional clues for understanding the immunosuppression or identifying the new therapeutic strategies for the management of immunosuppression.

## Author contributions

Conception and design of the research: Jin Chen and Jing Yan. Acquisition, analysis and interpretation of data: Fang Chen, Chunyan Yao, Yue Feng, Ying Yu, and Honggang Guo. Drafting the manuscript: Fang Chen and Chunyan Yao. Manuscript revision for important intellectual content: Yue Feng and Jing Yan. All authors have read and approved the manuscript.

**Conceptualization:** Fang Chen, JING YAN, Jin Chen.

**Data curation:** Fang Chen, Chunyan Yao, Yue Feng, Ying Yu, Honggang Guo.

**Formal analysis:** Fang Chen, Chunyan Yao, Yue Feng.

**Investigation:** Ying Yu, Honggang Guo, Jin Chen.

**Methodology:** Fang Chen, Chunyan Yao.

**Project administration:** JING YAN.

**Resources:** Fang Chen, Chunyan Yao, Yue Feng.

**Software:** Fang Chen, Chunyan Yao.

**Supervision:** JING YAN, Jin Chen.

**Validation:** Chunyan Yao, Honggang Guo.

**Writing – original draft:** Fang Chen.

**Writing – review & editing:** Yue Feng, JING YAN, Jin Chen.
